# Chronic stress induces NPD‐like behavior in APPPS1 and WT mice with subtle differences in gene expression

**DOI:** 10.1111/gbb.12766

**Published:** 2021-08-23

**Authors:** Amalie Clement, Mads M. Pedersen, Allan Stensballe, Ove Wiborg, Ayodeji A. Asuni

**Affiliations:** ^1^ Department of Health Science and Technology Aalborg University Aalborg Denmark; ^2^ Department of Pathology and Fluid Biomarkers H. Lundbeck A/S Copenhagen Denmark; ^3^ Department of Biostatistics H. Lundbeck A/S Copenhagen Denmark

**Keywords:** Alzheimer's disease, anxiety, chronic stress, HPA axis, neuropsychiatric disturbances, preclinical research model, sleep disturbances

## Abstract

Neuropsychiatric disturbances (NPDs) are considered hallmarks of Alzheimer's disease (AD). Nevertheless, treatment of these symptoms has proven difficult and development of safe and effective treatment options is hampered by the limited understanding of the underlying pathophysiology. Thus, robust preclinical models are needed to increase knowledge of NPDs in AD and develop testable hypotheses and novel treatment options. Abnormal activity of the hypothalamic–pituitary–adrenal (HPA) axis is implicated in many psychiatric symptoms and might contribute to both AD and NPDs development and progression. We aimed to establish a mechanistic preclinical model of NPD‐like behavior in the APPPS1 mouse model of AD and wildtype (WT) littermates. In APPPS1 and WT mice, we found that chronic stress increased anxiety‐like behavior and altered diurnal locomotor activity suggestive of sleep disturbances. Also, chronic stress activated the HPA axis, which, in WT mice, remained heightened for additional 3 weeks. Chronic stress caused irregular expression of circadian regulatory clock genes (BMAL1, PER2, CRY1 and CRY2) in both APPPS1 and WT mice. Interestingly, APPPS1 and WT mice responded differently to chronic stress in terms of expression of serotonergic markers (5‐HT_1A_ receptor and MAOA) and inflammatory genes (IL‐6, STAT3 and ADMA17). These findings indicate that, although the behavioral response to chronic stress might be similar, the neurobiochemical response was different in APPPS1 mice, which is an important insight in the efforts to develop safe and effective treatments options for NPDs in AD patients. Further work is needed to substantiate these findings.

## Graphical abstract

Our findings indicate that chronic stress exposure induce diurnal rhythm disruption together with clock gene dysregulation and increased anxiety in both APPPS1 mice and their wildtype (WT) littermates. Nevertheless, subtle differences were observed in the neuroinflammatory profile and serotonergic markers between APPPS1 and WT mice.

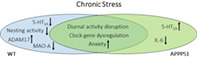

## 1 INTRODUCTION

Alzheimer's disease (AD) is one of the most prevalent neurodegenerative disorders causing both cognitive impairment and neuropsychiatric disturbances (NPDs). The 80%–97% of AD patients experience at least one NPD at least once during the course of their disease.^1^, ^2^ These behavioral changes can be single or reoccurring events or persist for longer periods but they rarely disappear.^3^ In AD patients, they are associated with worse quality of life, higher severity of disease,^4^ and increase caregiver burden, with apathy, anxiety and sleep disturbances having the highest impact on caregiver burden,^5^ while also being among the most prevalent NPDs in AD.^6^ NPDs have been shown to predict conversion from being cognitively normal to mild cognitive impairment (MCI) progression and from MCI to AD^7^, ^8^ together with a faster decline and death.^9^ Additionally, sleep disturbances, which can occur years before clinical AD,^10^, ^11^ have also been postulated as a risk factor for AD pathology development.^12^, ^13^ Nevertheless, pathological hallmarks of AD have been shown to appear years before clinical diagnosis^14^ and thus it is still uncertain if NPDs cause AD pathology or is rather a consequence of the pathological changes.

Although it is clear that NPDs play a significant role in the development of AD, recognizing and treating NPDs remains a major challenge due to the poor understanding of the underlying pathophysiology.^6^, ^15^ Treating NPDs with conventional mood modulating drugs in patients diagnosed with dementia has proven difficult with little or no effect over placebo but has been associated with an increased risk of adverse events and mortality.^16^, ^17^ This suggests, that although the disease symptomatology of NPDs share obvious similarities with well‐known symptoms of anxiety and depression, the causative neuro‐pathophysiology may be different. This underlines the unmet need for better understanding of the neurobiochemical changes of NPDs in order to develop safe and effective treatment options for AD patients experiencing NPDs. While cognitive impairment is well characterized in transgenic mouse models of AD,^18^ NPD‐like behavior is underrepresented in the literature.^19^ As we have previously stated^20^ the development of reliable mechanistic models of NPDs is a key limiting factor that needs to be addressed to support novel drug development efforts.

It is well documented that stress, and the activation of the HPA axis, is implicated in the regulation of mood,^21^ development of psychiatric disorders,^22^, ^23^, ^24^ AD^25^ and NPDs.^26^ AD patients show elevated levels of cortisol associating with a faster disease progression and cognitive decline.^27^, ^28^ Hyperactivation of the HPA axis, also known as allostatic overload, results in neuroinflammation,^25^ which is a classical hallmark of AD pathology and a contributing factor to psychiatric symptoms.^29^ Nevertheless, the synergistic effect of chronic stress, allostatic overload, neuroinflammation and NPDs on AD pathology is not fully understood.

In the present study, we aimed to establish a mechanistic model of NPD‐like behavior in a transgenic mouse model of AD (APPPS1) by introducing chronic stress. This model develops amyloidosis at 6–8 weeks with robust plaque formation and cognitive impairments at 7–8 months of age^30^, ^31^ but exhibit limited behavioral aspects of NPDs.^30^ Wildtype (WT) littermates were included in these studies to understand the chronic stress effects of the model in the absence of AD pathology. We found that our mechanistic chronic stress model displays a dysregulated HPA axis and NPD‐like behavioral changes in APPPS1 and WT mice. On the brain biochemical level, we found subtle differences in the expression of certain serotonergic and neuroinflammatory markers together with more robust changes in circadian regulatory genes. Although further investigations are needed to further substantiate these observations, we believe our findings support the validity of our chronic stress paradigm as a model for NPDs in AD and allow us to begin explaining the different response in AD patients to mood regulating drugs.

## 2 METHODS AND MATERIALS

### 2.1 Mouse model

The 6 months old male APPPS1 mice (expressing the human transgenes APP (Swedish [K670M and N671L] mutations) and PSEN1 (L166P mutation) on a C57BL/6J background strain^30^ and WT littermates purchased from Charles River (Germany) were used for these studies. Mice were housed individually upon arrival at the facility (10 weeks prior to study) and throughout the study period and received food and water ad libitum except for the deprivation periods during the chronic stress period. During the 10 weeks acclimatization period, mice were housed under standard lighting conditions (12 L/12D) with lights on at 6 am. Room humidity and temperature were 55% ± 5% and 21 ± 2°C, respectively. All experiments were in accordance with the European Communities Council Directive no. 86/609, the directives of the Danish National Committee on Animal Research Ethics, and Danish legislation on experimental animals (license no. 2014‐15‐0201‐00339 C01 and C05).

### 2.2 Chronic stress

**FIGURE 1:**
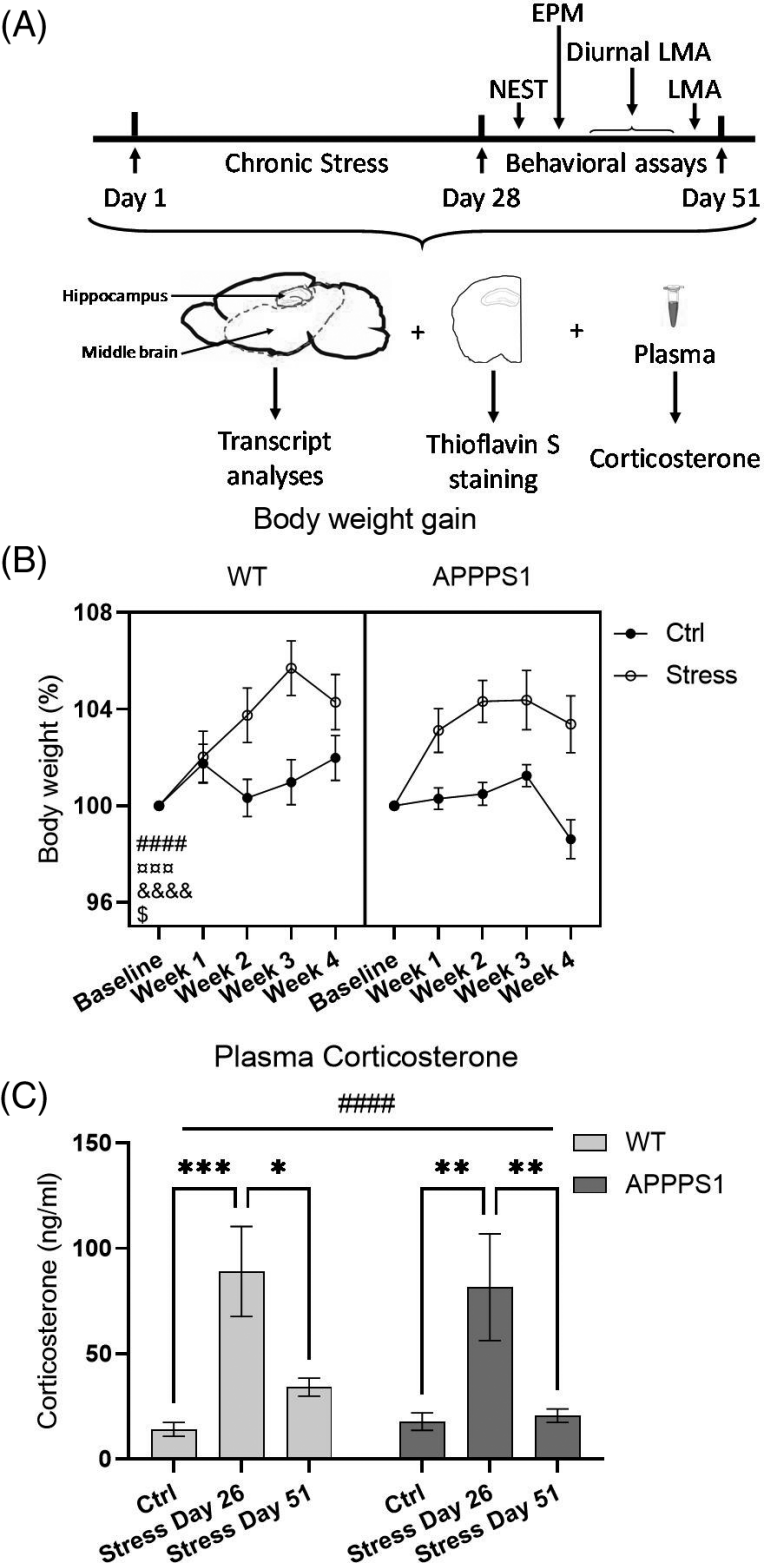
Chronic stress activates the hypothalamic–pituitary–adrenal (HPA) axis and increase body weight in WT and APPPS1 mice. (A) Schematic illustration of the experimental setup. Mice were subjected to 28 days of chronic stress followed by 23 days of behavioral assays and euthanization on day 51. (B) Weight gain was significantly affected by time (*p* < 0.0001, ####), stress (*p* = 0.0004, ¤¤¤), time × genotype (*p* = 0.0140, $), time × stress (*p* < 0.0001, &&&&) but not by genotype × stress. Three‐way ANOVA with repeated measures was used for statistical analysis. (C) Plasma was sampled on day 26 (ZT4) and day 51 (ZT5‐6) to analyze plasma corticosterone levels and the analysis showed a significant main effect of day (*p* < 0.0001, ####). Both APPPS1‐stress and WT‐Stress showed significantly elevations of plasma corticosterone on day 26 compared with APPPS1‐Ctrl (*p* = 0.0052, **) and WT‐Ctrl (*p* = 0.0008, ***), respectively. On Day 51, plasma corticosterone return to Ctrl levels in APPPS1‐stress and WT‐Stress mice. Statistical analysis was performed using two‐way ANOVA. Both graphs represent *n* = 13/group. Data represents mean ± *SEM*

Twenty‐six male WT and twenty‐six male APPPS1 mice were randomly assigned to either control (Ctrl) or chronic stress (Stress) groups, yielding four test groups of each 13 mice (WT‐Ctrl, WT‐Stress, APPPS1‐Ctrl and APPPS1‐Stress). The chronic stress protocol was inspired by^32^, ^33^, ^34^ and introduced one stressor per day together with diurnal disruption stress (10 h light and 10 h darkness [10 L/10D]). The stressors were as follows: Overnight (o.n.) food deprivation, o.n. water deprivation, o.n. cage tilt (35–40°) and 1‐hour confinement to small container (4,5 cm × 8 cm × 12 cm). Throughout the study period all mice were weighed weekly, but Ctrl mice were otherwise left with minimal disturbances. The chronic stress protocol ran from day 1 until day 28 followed by behavioral assays and euthanization on day 51 (Figure 1A). The diurnal disruption stressor (10 L/10D) was continued throughout the study period to avoid potential recovery due to a nonstressful environment. All mice were weighed weekly to monitor individual health status. On day 26 at zeitgeber time 4 (ZT4), cheek blood was sampled after the confinement stress exposure, by a trained technician, to assess HPA axis activation by plasma corticosterone levels. Cheek blood was sampled from Ctrl mice on the same day and timepoint.

### 2.3 Behavior

On day 31, the nest building assay was performed as previously described.^35^ Briefly, standard nesting material were replaced by a cotton nestlet (5 × 5 cm). The assay was initiated at ZT1 and nesting quality was scored every hour until ZT9 and again 24 h after assay initiation. The degree of shredded nestlet and coverage of igloo openings scored from 0 to a maximum score of 8 (complete shredding and coverage of all three igloo openings).

On day 36, elevated plus maze (EPM) was performed from ZT2 to ZT5. The assay were as previously described.^36^ Briefly, after a 30 min acclimatization, mice explored the maze freely for 5 min. Ethovision XT15 (Noldus Information Technology, Netherlands) was used for automatic scoring of time in open arms, time in closed arms, and number of head dips.

On day 41, diurnal locomotor activity (LMA) was initiated at ZT3 and ran for 72 h as described previously.^37^ Mice were placed in individual activity cages with food and water ad libitum. Activity cages were equipped with light beams and connected to the UMOTWin software (Ellegaard Systems, Denmark) for automatic scoring of LMA. On day 50, LMA and rearing was scored using the same UMOTWin software system. This assay was performed from ZT3 to ZT6.

### 2.4 Euthanization

Mice were brought to the euthanization room immediately before euthanization on day 51. Euthanization took place in the middle of the light phase (Stress mice ZT5‐ZT6, Ctrl mice ZT6‐ZT7) by awake decapitation and plasma and brains were obtained as previously described^38^ with the exception of left hemispheres, where hippocampus and middle brain were macro‐dissected (see schematic illustration in Figure 1A). Samples termed “middle brain” included the midbrain, cerebral nuclei and interbrain (which contains but is not limited to the hypothalamus and suprachiasmatic nucleus). All samples were snap frozen on dry ice and stored at −80°C.

### 2.5 Plasma corticosterone

Plasma corticosterone levels were determined using Corticosterone Enzyme‐Linked ImmunoSorbant Assay (ELISA) Kit (Arbor Assays Inc, USA) following manufacturer's instructions.

### 2.6 Reverse Transcription‐Polymerase Chain Reaction (RT‐PCR)

Middle brains and hippocampi were homogenized as previously reported.^39^ RNA was extracted using the NucloeSpin® RNA kit (Macherey‐Nagel). Reverse transcriptase was performed using iScript™ cDNA synthesis kit (Bio‐Rad) or TaqMan™ reverse transcription reagents (ThermoFisher) and 500 ng RNA per sample. PCR was performed using TaqMan™ Fast Advance mmix and TaqMan™ primers for the following genes: Gapdh (Mm99999915_g1), CRY1 (Mm00514392_m1), CRY2 (Mm01331539_m1), PER1 (Mm00501813_m1), PER2 (Mm00478099_m1), BMAL1 (Mm00500226_m1), CLOCK (Mm00455950_m1), IL‐6 (Mm00446190_m1), STAT3 (Mm00456961_m1), ADAM17 (Mm00456428_m1), GFAP (Mm01253033_m1), 5‐HT_1A_R (Mm00434106_s1) and MAO‐A (Mm00558004_m1). Target genes were normalized to Gapdh using the following formula: ∆Ct(target)‐∆Ct(Gapdh) = ∆∆Ct, and the relative expression changes were calculated as 2^(−∆∆Ct) of WT‐Ctrl.

### 2.7 Thioflavin S staining

Coronal brain sections (20 μm) from APPPS1 mice only were attached to Superfrost Plus glass (VWR, Denmark). To assess the level of plaque pathology, prefrontal cortex, isocortex and hippocampus were stained using Thioflavin‐S as previously described.^40^ Images were acquired on a Leica DM5500 B upright microscope equipped with Leica DFC450 camera using 2.5× object lens (Leica, Denmark). Quantification of plaques were performed using ImageJ.

### 2.8 Statistics

For these experiments, 13 animals per group were used (*n* = 13/group), unless otherwise stated in the Figure legends. All statistics and graph illustrations were performed using GraphPad Prism 9 and the statistical significance level was set to *p* ≤ 0.050. Two‐way ANOVA was chosen for statistical analyses of EPM, locomotor assay, plasma corticosterone, transcript analyses and light phase LMA with post hoc Bonferroni's correction for multiple comparisons. Three‐way ANOVA with repeated measures was used for the analysis of body weight and nesting behavior. To investigate a possible effect of time, time was included in this three‐way ANOVA as a factor with five or nine levels corresponding to the five or nine measured timepoints. All graphs show mean estimates and associated *SEM*. Significant main effects are illustrated with hashtag (#) while significant findings of post hoc multiple comparisons are illustrated with asterisk (*), unless otherwise stated in the figure legend.

## 3 RESULTS

Weekly body weighing was performed on all animals to monitor week‐by‐week effect of the stress procedures (Figure 1B). Three‐way ANOVA with repeated measures showed a significant source of variance of time (F_4,192_ = 17.22, *p* < 0.0001) and stress (F_1,48_ = 14.41, *p* = 0.0004) but not genotype (F_1,48_ = 0.5543, *p* = 0.4602). Additionally, we found a significant interaction of time × genotype (F_4,192_ = 3.210, *p* = 0.0140) and of time × stress (F_4,192_ = 9.719, *p* < 0.0001) but no interaction of stress x genotype. We also found a significant three‐way interaction of time × genotype × stress (F_4,192_ = 2.584, *p* = 0.0384). The stress‐induced weight gain could not be explained by altered expression of the hunger controlling satiety hormone leptin, as all groups show similar plasma leptin levels at day 26 (Figure S1).

Plasma samples drawn on day 26 (ZT4) and on day 51 (ZT5‐6) were used for determination of corticosterone levels. We found a significant effect of day (F_2,72_ = 14.38, *p* < 0.0001; Figure 1C) but no effect of genotype or interaction (genotype: F_2,72_ = 0.257, *p* = 0.613; interaction F_2,72_ = 0.1986, *p* = 0.820). Post hoc analyses showed significant elevation of plasma corticosterone on day 26 in both WT‐Stress and APPPS1‐Stress compared with WT‐Ctrl (*p* = 0.0008) and APPPS1‐Ctrl (*p* = 0.0052), respectively. However, plasma corticosterone returned to levels comparable to control levels on day 51 for APPPS1. Although the statistical analysis failed to show a significant change in plasma corticosterone levels on day 51, WT‐Stress gain a 142,45% higher mean plasma corticosterone level on day 51 compared with WT‐Ctrl with no overlap between 95% Confidence Interval (CI) of the two groups (WT‐Ctrl mean = 14.095 with 95% CI lower limit = 6.872 and upper limit = 21.317; WT‐Stress day 51 mean = 34.173 with 95% CI lower limit = 24.835 and upper limit = 43.511).

## 4 NPD‐LIKE BEHAVIOR

**FIGURE 2:**
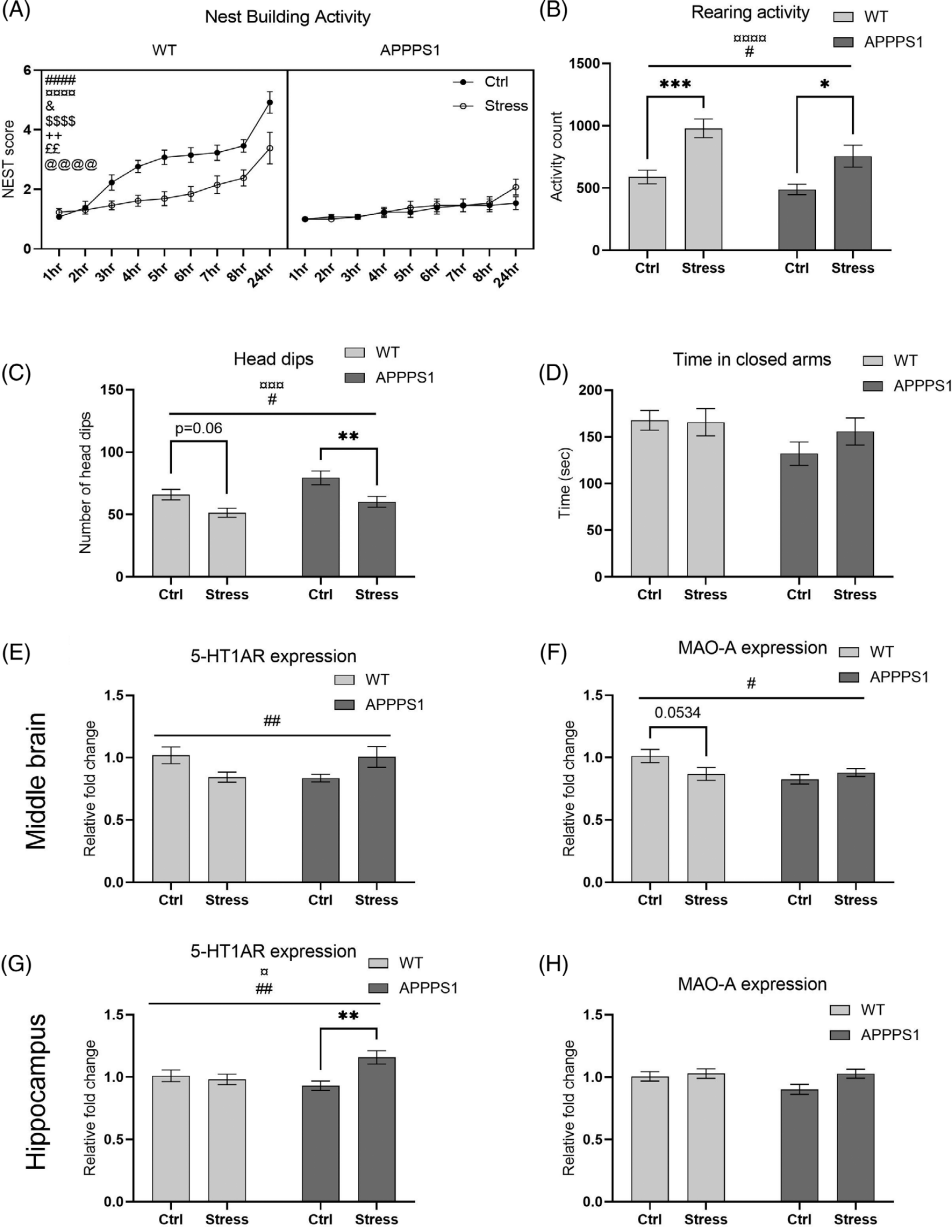
Chronic stress induces neuropsychiatric disturbances (NPD)‐like behavior and altered expression of 5‐HT1A‐R and MAO‐A in APPPS1 and WT mice. (A) Nest building activity were significantly affected by time (*p* < 0.0001, ####), genotype (*p* < 0.0001, ¤¤¤¤) and stress (*p* = 0.0131, &). Moreover, significant interaction effect were also observed of time × genotype (*p* < 0.0001, $$$$), time × stress (*p* = 0.0087, ++), genotype × stress (*p* = 0.0032, ££) together with a significant three‐way interaction of time x genotype × stress (*p* < 0.0001, @@@@). Nesting was performed with *n* = 13/group. (B) Rearing activity was significantly affected by stress (*p* = 0.0005, ¤¤¤¤) and by genotype (*p* = 0.0172, #). Rearing activity was assessed using *n* = 13 WT‐Ctrl, *n* = 8 WT‐Stress, *n* = 11 APPPS1‐Ctrl, and *n* = 8 APPPS1‐stress. (C) Chronic stress exposure reduced the number of head dips in both APPPS1 and WT mice, during the elevated plus maze (EPM) assay, illustrated by a significant main effect of stress (*p* = 0.0005, ¤¤¤) and of genotype (*p* = 0.0172, #). EPM was performed with *n* = 13/group except WT‐Ctrl where *n* = 11. (D) However, chronic stress exposure did not affect time spent in closed arms in the EPM. (E) There was a significant interaction of genotype x stress on the expression of 5‐HT1AR in the middle brain (*p* = 0.0065, ##). (G) Similar interaction effect was observation for the expression of 5‐HT1AR in hippocampus (*p* = 0.0077, ##) together with a main effect of stress (*p* = 0.0355, ¤) and post hoc significant difference between APPPS1‐Ctrl and APPPS1‐Stress (*p* = 0.0023, **). (F) Expression of MAO‐A in middle brain showed a significant interaction effect (p = 0.031, #), however post hoc analysis only showed a trend of decreased expression in WT‐Stress compared with WT‐Ctrl (*p* = 0.053). (H) MAO‐A expression was unaffected in the hippocampus. Nesting activity data was analyzed using three‐way ANOVA with repeated measures, while all other data was analyzed using two‐way ANOVA with post hoc Bonferroni's multiple comparisons. Expression analyses were performed using *n* = 9/group for middle brain and *n* = 10/group for hippocampus

The nest building assay is used to assess goal‐directed motivated behavior and we found a significant source of variance of time (F_2.863,137.4_ = 56.48, *p* < 0.0001; Figure 2A), genotype (F_1,48_ = 41.63, *p* < 0.0001) and stress (F_1,48_ = 6.634, *p* = 0.0131). Moreover, we found a significant interaction of time × genotype (F_8,384_ = 17.40, *p* < 0.0001), time × stress (F_8,384_ = 2.608, *p* = 0.0087) and genotype × stress (F_1,48_ = 9.651, *p* = 0.0032). Lastly, we also found a significant three‐way interaction of time x genotype x stress (F_8,384_ = 5.332, *p* < 0.0001). Altogether, this suggests that chronic stress exposure reduces nesting activity in WT mice and that APPPS1 mice are substantial impaired in this behavior independently of chronic stress.

Next, we used EPM to examine the effect of stress on anxiety‐like behavior. Even though we found no significant effect on time spent in closed arms (Figure 2D), we found a significant effect of genotype and stress on number of head dips (genotype F_1,46_ = 6.112, *p* = 0.0172; stress F_1,46_ = 13.95, *p* = 0.0005) with no corresponding interaction effect (F_1,46_ = 0.2736, *p* = 0.6035; Figure 2C). The post hoc multiple comparisons, with Bonferroni's corrections, showed a significant reduction of head dips in APPPS1‐Stress mice compared with APPPS1‐Ctrl (*p* = 0.0070) and a similar trend in WT‐Stress mice compared with WT‐Ctrl (*p* = 0.0623). Head dipping has been linked to exploratory behavior in rodents^41^ and anxiety decreases exploratory behavior,^42^ therefore we interpret reduction in head dips as a measure of anxiety‐like behavior induced by the chronic stress. Moreover, we found a significant effect of genotype (F_1,36_ = 6.350, *p* = 0.0163 Figure 2B) and stress (F_1,36_ = 26.40, *p* < 0.0001) on rearing activity with no significant effect of the corresponding interaction (F_1,36_ = 0.9277, *p* = 0.3419) suggesting that chronic stress affects rearing activity independently of genotype. Post hoc analysis showed a significant elevation of rearing activity in both APPPS1 and WT following chronic stress (*p* = 0.0125 and *p* = 0.0002, respectively). Unsupported rearing is highly linked to emotional state, while supported rearing is closer associated with locomotor activity.^43^ The experimental setup of the LMA assay used for these studies does not discriminate between supported and unsupported rearing. However, together with the reduced head dips in EPM, we would argue that the increased rearing activity might be associated with increased fearfulness and hyper alertness to the unfamiliar surroundings of the experimental LMA boxes.

The 5‐HT_1A_ receptor (5‐HT_1A_R) has been shown to be implicated in anxiety behavior and stress response^44^ and the monoamine oxidase A (MAO‐A), which degrade serotonin, has been shown to be increased in depression disorders and is regulated by the glucocorticoid receptor, which binds cortisol/corticosterone.^21^ For these reasons, we investigated the expression of these genes in hippocampus and middle brain tissue. Two‐way ANOVA illustrated no effect of genotype or stress, but a significant interaction of genotype x stress (F_1,32_ = 8.465, *p* = 0.0065; Figure 2E) on 5‐HT_1A_R expression in the middle brain, although post hoc analysis only showed a trend of change (APPPS1‐Stress vs. APPPS1‐Ctrl p = 0.103; WT‐Stress vs. WT‐Ctrl *p*  = 0.089). In hippocampus, we found a significant main effect of stress on the expression of 5‐HT_1A_R (F_1,36_ = 4.771, *p* = 0.0355; Figure 2G) with no effect of genotype (F_1,36_ = 1.152, *p* = 0.290) but a significant corresponding interaction (F_1,36_ = 7.964, *p* = 0.0077). Post hoc analysis showed a significant difference in expression of 5‐HT_1A_R in APPPS1 mice following chronic stress (*p* = 0.0023). Expression of MAO‐A in middle brain also resulted in a trend of main effect of genotype (F_1,32_ = 3.961, *p* = 0.0552; Figure 2F), no effect of stress (F_1,32_ = 1.056, *p* = 0.312) with a significant corresponding interaction (F_1,32_ = 5.100, *p* = 0.0309), while the post hoc analysis only showed a trend of difference between WT‐Ctrl and WT‐Stress (*p*= 0.0534). Expression of MAO‐A in hippocampus showed trend of significant effect of stress (F_1,36_ = 3.752, *p* = 0.0606; Figure 2H) with no effect of genotype (F_1,36_ = 1.912, *p* = 0.175) or corresponding interaction (F_1,36_ = 1.833, *p* = 0.184).

## 5 CIRCADIAN RHYTHM ALTERATIONS

**FIGURE 3:**
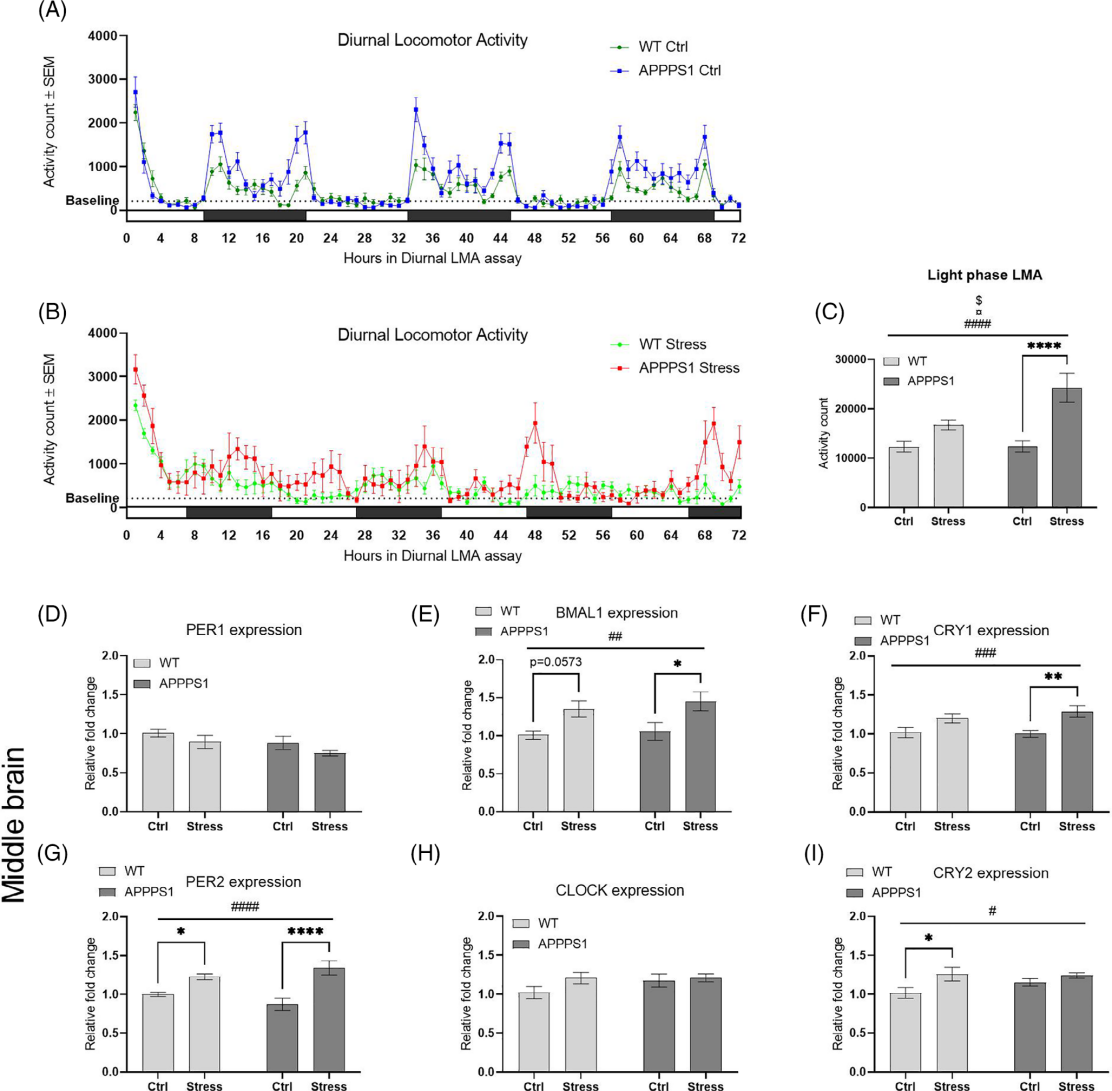
Chronic stress altered diurnal locomotor activity rhythm with abnormal expression of circadian regulatory genes. (A + B) Graphic illustration of the diurnal LMA under control conditions (A) and diurnal disruption conditions (B). Black bars indicate dark periods while white bars indicate light periods. The dotted line indicates a baseline LMA defined by the mean LMA of the WT‐Ctrl in light phase.( C) Light phase activity was significantly affected by stress (*p* < 0.0001, ####), genotype (*p* = 0.0195, ¤) and the corresponding interaction effect (*p* = 0.0209, $). Furthermore, post hoc analysis showed a significant difference between APPPS1‐Ctrl and APPPS1‐Stress (*p* < 0.0001, ****). Diurnal locomotor activity and light phase locomotor activity (LMA) was performed using *n* = 13/Ctrl group and *n* = 8/Stress group. Expression of circadian regulatory genes in middle brain was analyzed and fold change calculated relative to WT‐Ctrl mice. (E) Expression of BMAL1 was significantly affected by stress (*p* = 0.0014, ##) and post hoc analysis showed a significant difference between APPPS1‐Ctrl and APPPS1‐stress (*p* = 0.0243, *), and a trend of significant difference between WT‐Ctrl and WT‐Stress (*p* = 0.0573). (F) Likewise, expression of CRY1 was also significantly affected by stress (p = 0.0006, ###) together with a significant difference between APPPS1‐Ctrl and APPPS1‐Stress (*p* = 0.0049, **), and a trend of significant difference between WT‐Ctrl and WT‐Stress (*p* = 0.0885). (G) We observed similar significant main effect of stress on expression of PER2 (*p* < 0.0001, ####) and significant differences between APPPS1‐Ctrl and APPPS1‐Stress (*p* < 0.0001, ****) and between WT‐Ctrl and WT‐Stress (*p* = 0.0425, *). (I) Lastly, the same pattern was observed for expression of CRY2 with a significant main effect of stress (*p* = 0.0133, #) and a significant difference between WT‐Ctrl and WT‐Stress (*p* = 0.0212, *). No difference in expression levels were found for PER1 (D) and CLOCK (H) Statistical analyses were performed using two‐way ANOVA with post hoc Bonferroni's multiple comparisons. Expression analyses were performed using *n* = 9/group for middle brain and *n* = 10/group for hippocampus

Sleep disturbances are common in both AD^6^ and stress related disorders.^45^, ^46^ In this study we analyzed the diurnal LMA for 72 h as a measure of diurnal rhythm relating to sleep patterns. Figure 3A illustrates the diurnal LMA of APPPS1‐Ctrl and WT‐Ctrl with high activity during dark phases and low activity during light phases. This diurnal pattern is clearly changed with chronic stress exposure (Figure 3B). Indeed, we found a significant effect of both genotype (F_1,38_ = 5.950, *p* = 0.0195; Figure 3C) and stress (F_1,38_ = 27.54, *p* < 0.0001) together with a significant corresponding interaction (F_1,38_ = 5.812, *p* = 0.0209) on light phase LMA. Post hoc analysis showed that APPPS1‐Stress mice had significantly higher activity levels in light phase compared with APPPS1‐Ctrl (*p* = <0.0001; Figure 3C), which was only trending for WT‐Stress compared with WT‐Ctrl (*p* = 0.104). Mice are nocturnal and are mostly inactive and sleeping during the light phase, thus it is likely that the increased LMA in light phase points toward stress‐induced sleep disturbances. Supporting this notion, we found that chronic stress significantly altered expression of circadian regulatory genes. Stress had a significant main effect on the expression of BMAL1 in middle brain tissue, which included the hypothalamus and suprachiasmatic nucleus (F_1,32_ = 12.25, *p* = 0.0014; Figure 3E) with no effect of genotype (F_1,32_ = 0.527, *p* = 0.473) or interaction (F_1,32_ = 0.067, *p* = 0.797). The post hoc multiple comparisons, with Bonferroni's corrections, showed a significant increase of BMAL1 expression in APPPS1‐Stress compared with APPPS1‐Ctrl (*p* = 0.0243) with a trend of a similar increase in WT‐Stress compared with WT‐Ctrl (*p* = 0.057). Likewise, stress had a significant main effect on expression of PER2 in middle brain (F_1,32_ = 28.03, *p* < 0.0001; Figure 3G) with no effect of genotype (F_1,32_ = 0.01497, *p* = 0.903) but a trending corresponding interaction (F_1,32_ = 3.493, *p* = 0.0708). Post hoc analysis showed a significant increased expression of PER2 in both APPPS1 and WT following stress (*p* < 0.0001 and *p* = 0.0425, respectively). Expression of CRY1 also showed a significantly effect of stress (F_1,32_ = 14.49, *p* = 0.0006; Figure 3F) with no effect of genotype (F_1,32_ = 0.323, *p* = 0.574) or the corresponding interaction (F_1,32_ = 0.713, *p* = 0.405). The post hoc analysis showed a significant increased expression of CRY1 in APPPS1‐Stress compared with APPPS1‐Ctrl (*p* = 0.0049) with a similar trend in WT‐Stress compared with WT‐Ctrl (*p* = 0.089). Similarly, expression of CRY2 was significantly impacted by stress (F_1,32_ = 6.876, *p* = 0.0133; Figure 3I) with no effect of genotype (F_1,32_ = 0.860, *p* = 0.361) or corresponding interaction (F_1,32_ = 1.484, *p* = 0.232). Post hoc analysis showed a significant increase of CRY2 expression in WT mice following chronic stress (*p* = 0.0212) but not in APPPS1 mice (*p* = 0.657). Chronic stress appeared to have no effect on expression of PER1 and CLOCK in this cohort of animals (Figure 3D,H).

## 6 AMYLOID PATHOLOGY

Although chronic stress has been linked to the development of AD,^25^ we observed no changes in either plaque load or plaque count in prefrontal cortex, hippocampus or isocortex in APPPS1 mice following chronic stress (Figure S3). Therefore, the stress‐induced behavioral alterations observed in APPPS1‐Stress mice cannot be explained by accelerated β‐amyloid plaque pathology in the current study.

## 7 NEUROINFLAMMATION

Chronic stress has been linked to increased central^47^and peripheral^48^ inflammation, and the proinflammatory cytokine IL‐6 has been suggested as the predominant stress inducible cytokine.^49^ Therefore, we investigated the expression of IL‐6 together with genes related to IL‐6 signaling in hippocampus and middle brain following chronic stress.

**FIGURE 4:**
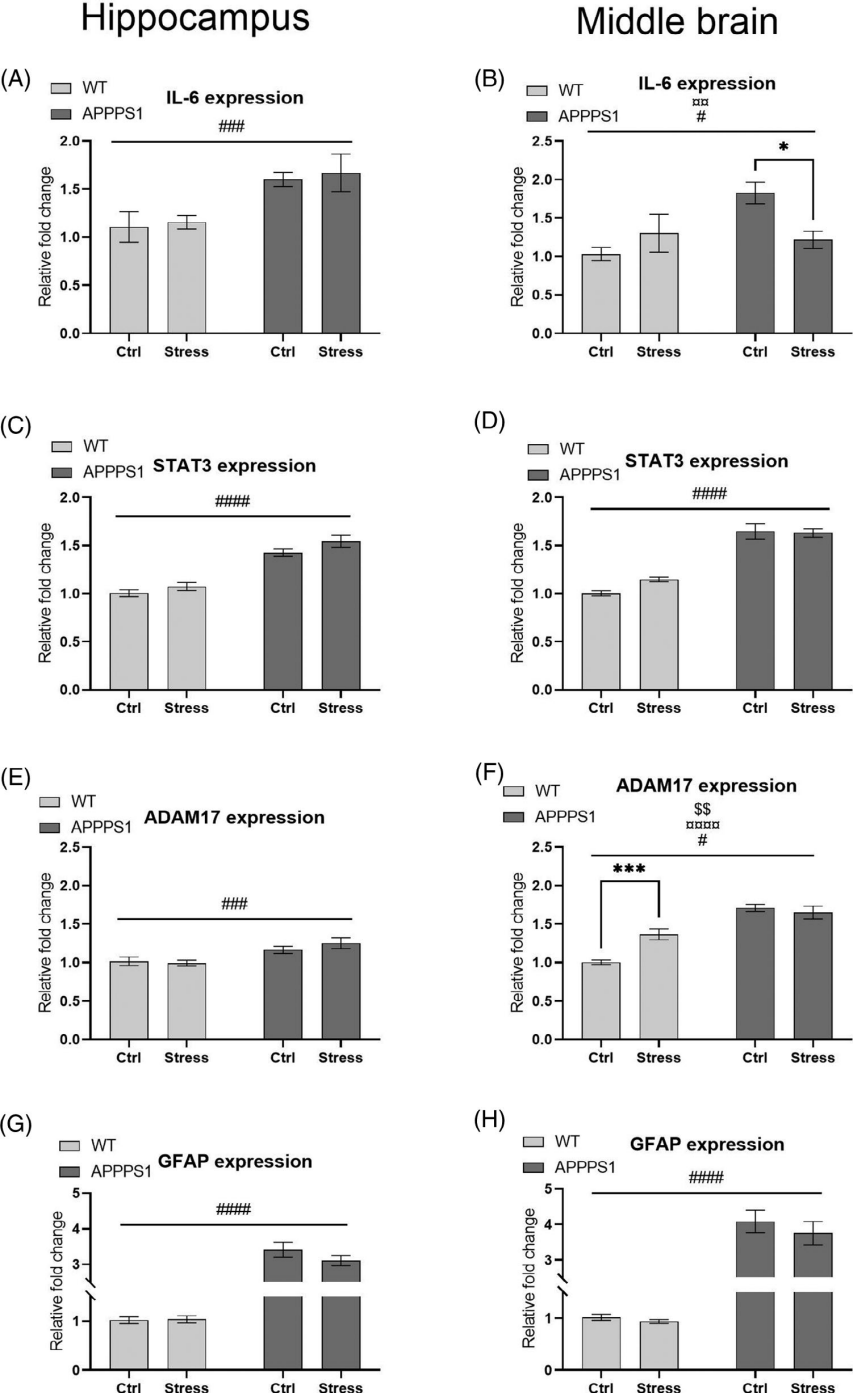
Chronic stress had subtle effect on IL‐6 dependent neuroinflammation in APPPS1 and WT mice middle brain but not in hippocampus. (A) Expression of IL‐6 in hippocampus was significantly affected by genotype (*p* = 0.0007, ###), while expression of IL‐6 in middle brain was significantly affected by genotype (*p* = 0.0314, #) and an interaction effect (*p* = 0.0090, ¤¤, B), while post hoc analysis showed a significant reduction in APPPS1‐Stress compared with APPPS1‐Ctrl (*p* = 0.0204, *). (C and D) Expression of STAT3 was significantly affected by genotype in hippocampus (p < 0.0001, ####, C) and in middle brain (*p* < 0.0001, ####, D). (E) Likewise, expression of ADAM17 was significantly affected by genotype in hippocampus (*p* = 0.0007, ###), (F) while expression of ADAM17 in middle brain was significantly affected by stress (*p* = 0.0203, #), genotype (p < 0.0001, ¤¤¤¤) and the interaction effect (*p* = 0.0017, $$). Furthermore, we observed a significant increase in expression of ADAM17 in the middle brain in WT‐Stress compared with WT‐Ctrl (*p* = 0.0005, ***). G and H) GFAP expression was significantly affected by genotype in both hippocampus (*p* < 0.0001, ####, G) and middle brain (*p* < 0.0001, ####, H). All graphs illustrate expression in fold change compared with WT‐Ctrl mice. Statistical analyses were performed using two‐way ANOVA with Bonferroni's post hoc multiple comparisons. Expression analyses were performed using *n* = 9/group for middle brain and *n* = 10/group for hippocampus

In hippocampus, we found, a significant effect of genotype on the expression of IL‐6 (F_1,36_ = 13.66, *p* = 0.0007; Figure 4A) with no effect of stress (F_1,36_ = 0.185, *p* = 0.668) or interaction (F_1,36_ = 0.006, *p* = 0.939). This suggests that chronic stress exposure had no or limited effect on the expression of IL‐6 in hippocampus of these mice, but that the transgenic expression of APP and PS1 increased IL‐6 expression. Nevertheless, in middle brain we found a significant effect of genotype (F_1,32_ = 5.066, *p* = 0.0314; Figure 4B) together with a significant interaction (F_1,32_ = 7.730, *p* = 0.009) on the expression of IL‐6. Here, the post hoc analysis showed a significant decrease of IL‐6 expression in APPPS1‐Stress compared with APPPS1‐Ctrl (*p* = 0.0204). Next, we analyzed expression of the IL‐6 activated transcription factor STAT3 and found a significant effect of genotype (F_1,36_ = 92.22, *p* < 0.0001; Figure 4C), a trending effect of stress (F_1,36_ = 4.052, *p* = 0.0516) and no significant interaction (F_1,36_ = 0.334, *p* = 0.573) in hippocampus. Again, we found a significant effect of genotype (F_1,32_ = 129.4, *p* < 0.0001; Figure 4D) on the expression of STAT3 in middle brain with no effect of stress (F_1,32_ = 1.774, *p* = 0.192) or interaction (F_1,32_ = 2.541, *p* = 0.121).

The enzyme ADAM17 cleaves membrane bound IL‐6 receptor (IL‐6R) to secrete the pro‐inflammatory soluble IL‐6R. In hippocampus, we found a significant effect of genotype (F_1,36_ = 13.70, *p* = 0.0007; Figure 4E) with no significant effect of stress (F_1,36_ = 0.304, *p* = 0.585) or an interaction (F_1,36_ = 1.022, *p* = 0,319). However, in middle brain the expression of ADAM17 was significantly impacted by genotype (F_1,32_ = 63.99, *p* < 0.0001; Figure 4F), stress (F_1,32_ = 5.961, *p* = 0.0203) and with a significant corresponding interaction (F_1,32_ = 11.68, *p* = 0.0017). Furthermore, post hoc analyses showed a significantly increased expression of ADAM17 in WT‐Stress compared with WT‐Ctrl (*p* = 0.0005). Lastly, we analyzed the expression of GFAP in both hippocampus and middle brain of these mice, as GFAP is a commonly used marker of astrocyte activation and thus neuroinflammation. In both tissue types expression of GFAP was significantly affected by genotype (hippocampus F_1,36_ = 262.4, *p* < 0.0001; Figure 4G; middle brain F_1,32_ = 160.6, *p* < 0.0001; Figure 4H) with no significant effect of stress (hippocampus F_1,36_ = 1.042, *p* = 0.314; middle brain F_1,32_ = 0.769, *p* = 0.387) or corresponding interaction (hippocampus F_1,36_ = 1.341, *p* = 0.255; middle brain F_1,32_ = 0.298, *p* = 0.589). Altogether, these transcript analyses illustrate a subtle effect of chronic stress on IL‐6 and ADAM17 in middle brain, although the predominate results illustrate a substantial increased neuroinflammatory profile in APPPS1 mice.

## 8 DISCUSSION

We present a preclinical mechanistic model of NPD‐like behavior in APPPS1 and WT mice with activation of the HPA axis, alterations of circadian clock gene transcription, neuroinflammation and serotonergic regulation. Although, further independent validation of our model is needed, we believe this work holds the potential to expand our understanding of sleep disturbances, apathy, anxiety and hypervigilance with and without AD neuropathology.

Apathy is a neurocognitive disturbance defined by reduced motivation combined with either reduced goal‐directed behavior, reduced goal‐directed cognitive activity or emotional flattening.^1^ Nest building activity in rodents is highly motivated, goal‐directed, innate behavior.^50^, ^51^ Therefore, we argue that the reduced nesting activity in WT‐Stress can be interpreted as apathy‐like behavior. Chronic stress exposure did not further reduce nesting impairments of APPPS1 mice, indicating that this apathy‐like phenotype was driven by the amyloidosis evident at 6 months of age.^30^ Others have found similar impaired nesting activity in AD transgenic models^52^, ^53^ and this is supported by clinical data showing an association between apathy and Aβ deposits in cortex^54^ and prefrontal cortex^55^ in MCI or AD patients.

We found abnormal diurnal LMA in both APPPS1 and WT mice following chronic stress exposure. Further analysis showed that this was driven by increased light phase activity, which could indicate altered sleep patterns. For this reason, we argue that the chronic stress paradigm induced sleep disturbances in APPPS1‐Stress mice with similar trends in the WT‐Stress group. The robust stress‐induced irregular clock gene expression in both genotypes further supports this notion. The light phase LMA was more pronounced in APPPS1‐Stress than WT‐Stress, underlining the importance of AD‐like neuropathology on diurnal activity patterns. Thus, it is likely that extensive amyloidosis leaves the brain more vulnerable to the stressors applied in our chronic stress model resulting in higher impact on circadian rhythms. This is in line with the reported bidirectional relationship between sleep disturbances and AD pathology^13^ and the high prevalence of sleep disturbances in AD.^6^

In addition to the above‐mentioned stress‐induced effects, suggestive of sleep disturbances, we found that chronic stress exposure significantly impacted body weight in both genotypes. A link between sleep disturbances and obesity have previously been suggested in humans^56^ and jet‐lag models have illustrated strong body weight gain in mice with no change in food intake.^57^, ^58^ Because plasma leptin levels were unchanged with chronic stress exposure (Figure S1), we expect no change in food intake and therefore this cannot explain the weight gain. We have previously found that diurnal disruption stress increased body weight in 6 months old APPPS1 with no change in food intake (Figure S4). Weight loss or reduced weight gain following the chronic mild stress (CMS) model has been reported extensively.^59^ However, to the best of our knowledge, this is the first time that a multiple stressors model has been combined with a diurnal stress model of 10 L/10D. The stress‐effect on weight was strongest from baseline until week 2–3, after which the effect plateaued/reduced. This first response is in line with our previous work and other reports,^57^, ^58^ while the plateaued/reduced weight gain response in the last week(s) more closely resembles reports on the CMS model. It is possible that 10 L/10D actives one set of metabolic pathways, causing weight gain, while exposure to multiple stressors (food/water deprivation, cage tilt, confinement) activates a different set of metabolic pathways, causing weight loss.

The diurnal LMA assay showed a prolonged acclimatization period in both APPPS1‐Stress and WT‐Stress, which might indicate increased alertness to the surroundings similar to hypervigilance, a state of high alertness to potential threats by excessively scanning the surroundings.^60^ To understand this better we defined a baseline activity as the mean of light phase activity count of WT‐Ctrl (= 263.4). Both APPPS1‐Ctrl and WT‐Ctrl reach baseline activity after approx. After 4–5 h, while WT‐Stress and APPPS1‐Stress reached baseline approx. Nineteen hours and 26 h after initiation, respectively. Generally, these mice spent little time at the baseline cutoff, suggesting a higher awareness to the surroundings. Hypervigilance is closely related to anxiety disorders and PTSD and extreme or chronic stress exposure has been suggested to trigger hypervigilance in humans^61^, ^62^ and animal models.^63^ Additionally, we found increased rearing activity following chronic stress exposure without altered LMA levels (Figure S2), which suggest increased scanning of surroundings and thus support the notion of stress‐induced hypervigilance in this study. Likewise, repeated restraint stress was reported to increase rearing activity in the open field test.^64^ Altogether, we interpret these behavioral findings as subtle indications of anxiety‐like behavior induced by the chronic stress.

We did not find a stress‐induced shift toward more time spent in closed compared with open arms during the EPM, which is generally considered as anxious behavior in rodents. However, the arms of the EPM apparatus, used in this study, measured 45 cm x 10 cm, which might be on the wide end for mouse studies.^65^ It is possible that the larger width has led to a lower anxiogenic effect of the open elevated space. Nevertheless, this would need to be studied further.

The 5‐HT_1A_‐R is involved in anxiety behavior and stress response.^44^ Our transcription analyses showed significantly increased expression of 5‐HT_1A_‐R in hippocampus of APPPS1‐Stress mice. Such stress‐induced elevated 5‐HT_1A_‐R levels have been reported previously.^66^, ^67^ Although only trending, MAO‐A expression in hippocampus of APPPS1‐Stress was higher than that of APPPS1 Ctrl, which might have caused lower levels of 5‐HT availability. Similar findings have been reported elsewhere.^68^ With the increased expression of both the inhibitory 5‐HT_1A_‐R and the 5‐HT degrading MAO‐A enzyme in hippocampus of APPPS1‐Stress, it is likely that the overall serotonergic tone was decreased and may account for the changed behavioral phenotype in these mice.

Furthermore, our results show that chronic stress had a different effect on the serotonergic system in APPPS1 mice than in WT mice. This difference may start to explain the current challenges of treating NPDs in AD patients.^16^, ^17^ Additionally, it underlines the relationship between stress, behavior and the serotonergic system, which has also been shown in treatment paradigms with the antidepressant vortioxetine, which can prevent stress‐induced learning impairments in rats^69^ and promote neurogenesis by enhancing glucocorticoid receptor response to acute stress in the ventral hippocampus.^70^ Vortioxetine is a serotonin transporter inhibitor and agonist of 5‐HT_1A_‐R among other 5‐HT receptor subtypes.^71^

Although NPDs are associated with faster decline in AD,^72^ we did not find changes in Aβ pathology in relation to stress‐induced NPD‐like behavior. NPDs can appear in the prodromal phase of AD^73^ and might predict severity of decline.^9^ Thus, one possible explanation for the missing stress‐effect on Aβ pathology could be the age of mice used in this study. Another explanation might be that NPD‐like behavior needs to be present for a longer period in order to affect plaque pathology. One research group found that chronic stress accelerated Aβ pathology in the Tg2576 model,^74^ while another group failed to show this effect in the TgCRND8 model.^75^

The analysis of the neuroinflammatory profile only found a significant effect of genotype on expression of IL‐6, STAT3, ADAM17 and GFAP in hippocampus and of STAT3 and GFAP in middle brain. This illustrates a high level of neuroinflammation in APPPS1 mice, which was unaffected by chronic stress. APPPS1 mice exhibit extensive microgliosis and astrocytosis at 8 months of age,^30^ and it is possible that any pro‐inflammatory effect of stress might have been too small to catch at that age. This notion can be substantiated in APPPS1 mice at ages prior to overt amyloid depositions. ADAM17 is a metalloprotease that cleaves IL‐6 receptor to secrete soluble IL‐6 receptor, which can activate the pro‐inflammatory trans‐signaling pathway via binding of gp130.^76^ Expression of ADAM17 was highly increased in WT‐Stress middle brain compared with WT‐Ctrl, which might have resulted in more soluble IL‐6‐R and activation of the trans‐signaling pathway even though IL‐6 expression levels were unchanged.

IL‐6 expression was significantly reduced in middle brain of APPPS1‐Stress. Many studies have found that chronic stress increase IL‐6 protein and/or mRNA levels,^48^, ^77^, ^78^ however, all measurements were in close relation to last stress‐exposure. Nevertheless, Voorhees and colleagues showed that IL‐6 mRNA levels were unchanged in hippocampus following chronic stress, both measured in close proximity to last stress exposure and in the recovery period.^79^ We speculate that a potential stress‐effect on IL‐6 expression might have recovered during the behavioral testing period and caused a hypo‐expression in APPPS1 Stress mice. Further studies are needed to substantiate this speculation.

## 9 LIMITATIONS

This study has some limitations and one of them is the lack of female mice used. Although AD is more prevalent in women than men,^80^ a trait that is also seen in depression and anxiety, we opted to use males for two main reasons: 1) The day to day handling of animals during the chronic stress protocol and behavioral assays was time consuming and excluding a gender would allow for a higher number of animals per group, and 2) two genders would add an independent factor to the statistical analyses resulting in more complex analyses.

Another limitation of this study is the potential recovery from day 28 to day 51 where APPPS1‐Stress and WT‐Stress were only exposed to the diurnal disruption stress (10 L/10D). Although we found stress‐induced alterations in gene expression at day 51 it is possible that these alterations would have been different at day 28.

Lastly, we did not include multiple behavioral assays for each disturbance (e.g. anxiety), which leaves the results slightly more subtle, and all memory/cognitive assays were left out although a clear relationship between mood and cognition have been reported.^81^

## 10 CONCLUSION

Here, we present a mechanistic preclinical model of chronic stress that induces NPD‐like behavior in the APPPS1 transgenic mouse model of AD and the corresponding WT littermates. Although, APPPS1‐Stress and WT‐Stress mice showed a comparable behavioral response to chronic stress, we observed subtle differences in gene expression of serotonergic and inflammatory markers, which might be valuable knowledge on the path to develop novel safe and effective treatment options for AD patients suffering from NPDs. Further investigations into these different responses in the AD‐like pathologic brain might pave the way to a novel piece of evidence to understand the neuropathophysiological of NPDs in AD.

## Supporting information


**Figure S1** Chronic stress did not change plasma leptin levels (interaction F_1,31_ = 0.06575, p = 0.7993; genotype F_1,31_ = 0.3943, p = 0.8473; stress F_1,31_ = 0.03769, p = 0.5347; n = 7‐10/group). Statistical analyses were performed using two‐way ANOVA. Data represents mean ± SEM.
**Figure S2**: Chronic stress did not change locomotor activity in APPPS1 or WT mice (stress condition F_1,37_ = 0.03397, p = 0.8548; genotype F_1,37_ = 3.706, p = 0.0619; interaction F_1,37_ = 0.2325, p = 0.6325). Statistical analysis was performed using two‐way ANOVA. n = 8‐13/group.
**Figure S3**: Thioflavin‐S staining of prefrontal cortex, hippocampus and isocortex in APPPS1 mice. We found no changes in plaque count (A + B + C) or plaque load (D + E + F) following exposure to chronic stress in any of the examined areas. Statistical analyses were performed using unpaired t‐test.
**Figure S4**: Body weight gain drastically increased in APPPS1 exposed to circadian disruption stress. 6 months old APPPS1 mice subjected to circadian disruption stress (dCc; 10 L/10D) or kept under normal circadian cycle (nCc; 12 L/12D). A) APPPS1 dCc mice showed a drastic increase in body weight gain compared to APPPS1 nCc (Time: F_2.748,145.6_ = 96.85, p < 0.0001; jet‐lag F_1,53_ = 19.91, p < 0.0001; time x jet‐lag F4,212 = 10.88 p < 0.0001; post hoc multiple comparison with Bonferroni's correction week 4 p = 0.0143, week 6 p < 0.0001, week 8 p = 0.0003), which could not be explained by an increase in food intake (p = 0.2900; B). Statistical analyses were performed using 2‐way ANOVA with repeated measures and post hoc multiple comparison with Bonferroni's correction (A) and unpaired t‐test (B).

## Data Availability

All data can be made available upon request.
