# Supplementary material for: Chronic stress induces NPD‐like behavior in APPPS1 and WT mice with subtle differences in gene expression
Source: Genes Brain Behav. 2021 Aug 23;20(8):e12766. doi: 10.1111/gbb.12766 (PMC9285501; doi:10.1111/gbb.12766)
Supplement: Supplementary file 1 — Figure S1 Chronic stress did not change plasma leptin levels (interaction F1,31 = 0.06575, p = 0.7993; genotype F1,31 = 0.3943, p = 0.8473; stress F1,31 = 0.03769, p = 0.5347; n = 7‐10/group). Statistical analyses were performed using two‐way ANOVA. Data represents mean ± SEM. Figure S2: Chronic stress did not change locomotor activity in APPPS1 or WT mice (stress condition F1,37 = 0.03397, p = 0.8548; genotype F1,37 = 3.706, p = 0.0619; interaction F1,37 = 0.2325, p = 0.6325). Statistical analysis was performed using two‐way ANOVA. n = 8‐13/group. Figure S3: Thioflavin‐S staining of prefrontal cortex, hippocampus and isocortex in APPPS1 mice. We found no changes in plaque count (A + B + C) or plaque load (D + E + F) following exposure to chronic stress in any of the examined areas. Statistical analyses were performed using unpaired t‐test. Figure S4: Body weight gain drastically increased in APPPS1 exposed to circadian disruption stress. 6 months old APPPS1 mice subjected to circadian disruption stress (dCc; 10 L/10D) or kept under normal circadian cycle (nCc; 12 L/12D). A) APPPS1 dCc mice showed a drastic increase in body weight gain compared to APPPS1 nCc (Time: F2.748,145.6 = 96.85, p < 0.0001; jet‐lag F1,53 = 19.91, p < 0.0001; time x jet‐lag F4,212 = 10.88 p < 0.0001; post hoc multiple comparison with Bonferroni's correction week 4 p = 0.0143, week 6 p < 0.0001, week 8 p = 0.0003), which could not be explained by an increase in food intake (p = 0.2900; B). Statistical analyses were performed using 2‐way ANOVA with repeated measures and post hoc multiple comparison with Bonferroni's correction (A) and unpaired t‐test (B). [file GBB-20-0-s001.docx]

**Supplementary material**

Supplementary Figure 1: Chronic stress did not change plasma leptin levels (interaction F_1,31_=0.06575, p=0.7993; genotype F_1,31_=0.3943, p=0.8473; stress F_1,31_=0.03769, p=0.5347; n=7-10/group). Statistical analyses were performed using two-way ANOVA. Data represents mean ± SEM.

Supplementary Figure 2: Chronic stress did not change locomotor activity in APPPS1 or WT mice (stress condition F_1,37_=0.03397, p=0.8548; genotype F_1,37_=3.706, p=0.0619; interaction F_1,37_=0.2325, p=0.6325). Statistical analysis was performed using two-way ANOVA. n=8-13/group.

Supplementary Figure 3: Thioflavin-S staining of prefrontal cortex, hippocampus and isocortex in APPPS1 mice. We found no changes in plaque count (A+B+C) or plaque load (D+E+F) following exposure to chronic stress in any of the examined areas. Statistical analyses were performed using unpaired t-test.

Supplementary Figure 4: Body weight gain drastically increased in APPPS1 exposed to circadian disruption stress. 6 months old APPPS1 mice subjected to circadian disruption stress (dCc; 10L/10D) or kept under normal circadian cycle (nCc; 12L/12D). A) APPPS1 dCc mice showed a drastic increase in body weight gain compared to APPPS1 nCc (Time: F_2.748,145.6_=96.85, p<0.0001; jet-lag F_1,53_=19.91, p<0.0001; time x jet-lag F4,212=10.88 p<0.0001; post hoc multiple comparison with Bonferroni’s correction week 4 p=0.0143, week 6 p<0.0001, week 8 p=0.0003), which could not be explained by an increase in food intake (p=0.2900; B). Statistical analyses were performed using 2-way ANOVA with repeated measures and post hoc multiple comparison with Bonferroni’s correction (A) and unpaired t-test (B).
